# Supplementary material for: Polyhydroxyalkanoate Production by Caenibius tardaugens from Steroidal Endocrine Disruptors
Source: Microorganisms. 2022 Mar 24;10(4):706. doi: 10.3390/microorganisms10040706 (PMC9027588; doi:10.3390/microorganisms10040706)
Supplement: Supplementary file 1 [file microorganisms-10-00706-s001.zip › microorganisms-1614208-supplementary.pdf]

Table S1. In silico identification of genes involved in PHA metabolism in *C. tardaugens* NBRC 16725 (accession number CP034179). The induction values (FC) of the genes upon culturing the bacteria in the presence of E2 and TES versus pyruvate (PYR) are included. RecA and GyrB encoding genes are included as housekeeping controls.

C. tardaugens		**FC**	
**Function**	**Gen (*EGO55_*)**	**TES vs PYR**	**E2 vs PYR**
β-ketothiolase (PhaA)	*09255* (*bktB*)	3.62	2.28
	20150	3.09	1.14
	05760	-4.39	-2.27
	05015	-1.30	1.60
	13750	4.92	2.80
	13790	2.21	1.77
	05665	-2.06	-1.63
	03475	-2.55	1.06
	14320	-1.91	-1.69
acetoacetyl-CoA reductase (PhaB)	*18540*	-1.31	-2.64
	09490	-2.25	-1.64
PHB synthase (PhaC)	*12105*	1.70	-1.04
	17580	1.09	-1.46
	18530	1.94	-1.01
PHB depolymerase (PhaZ)	*09590*	3.11	1.11
phasin (PhaP)	*12100*	34.08	18.05
housekeeping (RecA)	*01665*	2.11	1.38
housekeeping (GyrB)	*09795*	1.29	1.00

Table S2. In silico identification of genes related to PHA metabolism in bacteria belonging to the Sphingomonadaceae family described as PHA producers. Genes encoding homologous proteins and percentage identity to those from *C. tardaugens* found in genomes of *Sphingopyxis alaskensis* LMG 18877 (Godoy et al., 2003), *Sphingopyxis* chilensis S37 (Godoy et al., 2003) and *Novosphingobium nitrogenifigens* Y88^{T} (Smit et al., 2012) are included.

C. tardaugens				
**function**	**Gen (*EGO55_*)**	**organism**	**gen**	**% ID**
β-ketothiolase (PhaA)	*09255*	*S. chilensis* S37	-	-
	(bktB)	*S. alaskensis* LMG 18877	*Sala_2896*	43.80
		N. nitrogenifigens Y88	*Y88_0137*	58.93
	20150	*S. chilensis* S37	-	-
		S. alaskensis LMG 18877	*Sala_0781*	74.68
		N. nitrogenifigens Y88	*Y88_0992*	77.89
	05760	*S. chilensis* S37	-	-
		S. alaskensis LMG 18877	*Sala_2896*	44.67
		N. nitrogenifigens Y88	*Y88_3817*	71.82
	05015	*S. chilensis* S37	-	-
		S. alaskensis LMG 18877	*Sala_2162*	65.39
		N. nitrogenifigens Y88	*Y88_2522*	70.08
	13750	*S. chilensis* S37	-	-
		S. alaskensis LMG 18877	*Sala_3158*	53.06
		N. nitrogenifigens Y88	*Y88_3600*	53.18

	13790	*S. chilensis* S37	-	-
		S. alaskensis LMG 18877	*Sala_2162*	55.14
		N. nitrogenifigens Y88	*Y88_2522*	52.63
	05665	*S. chilensis* S37	-	-
		S. alaskensis LMG 18877	*Sala_1244*	45.43
		N. nitrogenifigens Y88	*Y88_0475*	39.64
	03475	*S. chilensis* S37	-	-
		S. alaskensis LMG 18877	*Sala_1637*	71.46
		N. nitrogenifigens Y88	*Y88_0475*	36.93
	14320	*S. chilensis* S37	-	-
		S. alaskensis LMG 18877	*Sala_1244*	44.08
		N. nitrogenifigens Y88	*Y88_0475*	39.41
acetoacetyl-CoA reductase (PhaB)	*18540*	*S. chilensis* S37	-	-
		S. alaskensis LMG 18877	*Sala_2255*	53.56
		N. nitrogenifigens Y88	*Y88_3538*	53.97
	09490	*S. chilensis* S37	-	-
		S. alaskensis LMG 18877	*Sala_2255*	79.58
		N. nitrogenifigens Y88	*Y88_3538*	80.00
PHB synthase (PhaC)	*12105*	*S. chilensis* S37	-	-
		S. alaskensis LMG 18877	*Sala_0505*	55.89
		N. nitrogenifigens Y88	*Y88_3019*	52.03
	17580	*S. chilensis* S37	-	-
		S. alaskensis LMG 18877	*Sala_0505*	37.40
		N. nitrogenifigens Y88	*Y88_2357*	68.73
	18530	*S. chilensis* S37	-	-
		S. alaskensis LMG 18877	*Sala_0505*	35.07
		N. nitrogenifigens Y88	*Y88_2357*	66.14
Phasin (PhaP)	*12100*	*S. chilensis* S37	-	-
		S. alaskensis LMG 18877	*Sala_0504*	50.83
		N. nitrogenifigens Y88	*Y88_3020*	62.18
PHB depolymerase (PhaZ)	*09590*	*S. chilensis* S37	-	-
		S. alaskensis LMG 18877	*Sala_1984*	66.17
		N. nitrogenifigens Y88	*Y88_2997*	67.89
PHB oligomer hidrolase (PhaY)	*15075*	*S. chilensis* S37	-	-
		S. alaskensis LMG 18877	*Sala_0774*	50.70
		N. nitrogenifigens Y88	*Y88_2567*	57.80

Table S3. In silico identification of genes related to PHA metabolism in different estrogen-degrading bacteria. Genes encoding homologous proteins and percentage identity to those from *C. tardaugens* found in genomes of *Croceicoccus estronivorus*, *Sphingomonas* sp. KC8, *Vibrio* sp. H5, *Achromobacter xylosoxidans* NBRC 15126, *Steroidobacter denitrificans* FST, *Denitratisoma oestradiolicum* DSM 16959, *Sphingobium estronivorans* AXB and *Sphingobium bisphenolivorans* YL23 are included.

C. tardaugens				
**function**	**Gen** *EGO55_*	**organism**	**gen**	**% ID**
β-ketothiolase (PhaA)	*09255* (bktB)	*C. estronivorus*	*MB02_08935*	87.69
		Sphingomonas sp. KC8	*KC8_00885*	48.72
		Vibrio sp. H5	-	-
		A. xylosoxidans NBRC 15126	*AX27061_4634*	59.18
		Sdo. denitrificans FST	*ACG33_03505*	43.40

	D. oestradiolicum DSM 16959	*CBW56_06210*	57.44
	S. estronivorans AXB	*F7P65_RS13585*	65.81
	S. bisphenolivorans YL23	*L858_RS0111020*	46.68
20150	*C. estronivorus*	*MB02_13535*	86.43
	Sphingomonas sp. KC8	*KC8_04605*	70.00
	Vibrio sp. H5	-	-
	A. xylosoxidans NBRC 15126	*AX27061_0119*	62.66
	Sdo. denitrificans FST	*ACG33_06440*	58.40
	D. oestradiolicum DSM 16959	*CBW56_06210*	39.75
	S. estronivorans AXB	*F7P65_RS09775*	69.39
	S. bisphenolivorans YL23	*L858_RS0114315*	71.10
05760	*C. estronivorus*	*MB02_09945*	44.42
	Sphingomonas sp. KC8	*KC8_00885*	45.61
	Vibrio sp. H5	-	-
	A. xylosoxidans NBRC 15126	*AX27061_3894*	70.35
	Sdo. denitrificans FST	*ACG33_03505*	45.00
	D. oestradiolicum DSM 16959	*CBW56_06210*	46.04
	S. estronivorans AXB	*F7P65_RS16970*	86.25
	S. bisphenolivorans YL23	*L858_RS0111020*	43.86
05015	*C. estronivorus*	*MB02_07390*	61.75
	Sphingomonas sp. KC8	*KC8_18205*	78.52
	Vibrio sp. H5	-	-
	A. xylosoxidans NBRC 15126	*AX27061_3311*	61.48
	Sdo. denitrificans FST	*ACG33_00745*	63.00
	D. oestradiolicum DSM 16959	*CBW56_04335*	56.75
	S. estronivorans AXB	*F7P65_RS17445*	81.54
	S. bisphenolivorans YL23	*L858_RS0111020*	38.75
13750	*C. estronivorus*	*MB02_15950*	84.64
	Sphingomonas sp. KC8	*KC8_01045*	69.27
	Vibrio sp. H5	-	-
	A. xylosoxidans NBRC 15126	*AX27061_3894*	41.65
	Sdo. denitrificans FST	*ACG33_00350*	63.38
	D. oestradiolicum DSM 16959	*CBW56_04315*	62.08
	S. estronivorans AXB	*F7P65_RS18190*	84.38
	S. bisphenolivorans YL23	*L858_RS0109310*	38.61
13790	*C. estronivorus*	*MB02_15915*	92.25
	Sphingomonas sp. KC8	*KC8_01010*	73.75
	Vibrio sp. H5	-	-
	A. xylosoxidans NBRC 15126	*AX27061_3311*	53.94
	Sdo. denitrificans FST	*ACG33_00745*	55.14
	D. oestradiolicum DSM 16959	*CBW56_04335*	64.91
	S. estronivorans AXB	*F7P65_RS18235*	86.50
	S. bisphenolivorans YL23	*L858_RS0111020*	39.23
05665	*C. estronivorus*	*MB02_10175*	37.38
	Sphingomonas sp. KC8	*KC8_06310*	63.16
	Vibrio sp. H5	-	-
	A. xylosoxidans NBRC 15126	*AX27061_3894*	40.86
	Sdo. denitrificans FST	*ACG33_04450*	54.27
	D. oestradiolicum DSM 16959	*CBW56_17310*	60.45
	S. estronivorans AXB	*F7P65_RS03380*	69.08
	S. bisphenolivorans YL23	*L858_RS0109310*	68.83

	03475	*C. estronivorus*	*MB02_10175*	41.23
		Sphingomonas sp. KC8	*KC8_06310*	45.91
		Vibrio sp. H5	-	-
		A. xylosoxidans NBRC 15126	*AX27061_4634*	38.02
		Sdo. denitrificans FST	*ACG33_04450*	48.09
		D. oestradiolicum DSM 16959	*CBW56_17310*	45.18
		S. estronivorans AXB	*F7P65_RS16205*	46.15
		S. bisphenolivorans YL23	*L858_RS0109310*	44.84
	14320	*C. estronivorus*	*MB02_10115*	35.59
		Sphingomonas sp. KC8	*KC8_06310*	69.17
		Vibrio sp. H5	-	-
		A. xylosoxidans NBRC 15126	*AX27061_4634*	36.90
		Sdo. denitrificans FST	*ACG33_04450*	54.41
		D. oestradiolicum DSM 16959	*CBW56_17310*	57.71
		S. estronivorans AXB	*F7P65_RS16205*	69.92
		S. bisphenolivorans YL23	*L858_RS0109310*	62.09
acetoacetyl-CoA reductase (PhaB)	*18540*	*C. estronivorus*	*MB02_08675*	53.14
		Sphingomonas sp. KC8	*KC8_08570*	56.07
		Vibrio sp. H5	-	-
		A. xylosoxidans NBRC 15126	*AX27061_1571*	50.00
		Sdo. denitrificans FST	*ACG33_13100*	50.42
		D. oestradiolicum DSM 16959	*CBW56_05405*	53.11
		S. estronivorans AXB	*F7P65_RS10460*	54.39
		S. bisphenolivorans YL23	*L858_RS0109895*	54.81
	09490	*C. estronivorus*	*MB02_08675*	90.42
		Sphingomonas sp. KC8	*KC8_08570*	78.33
		Vibrio sp. H5	-	-
		A. xylosoxidans NBRC 15126	*AX27061_5428*	49.18
		Sdo. denitrificans FST	*ACG33_13100*	50.00
		D. oestradiolicum DSM 16959	*CBW56_05400*	53.25
		S. estronivorans AXB	*F7P65_RS10460*	77.08
		S. bisphenolivorans YL23	*L858_RS0109895*	76.67
PHB synthase (PhaC)	*12105*	*C. estronivorus*	*MB02_01745*	78.75
		Sphingomonas sp. KC8	*KC8_11780*	61.17
		Vibrio sp. H5	-	-
		A. xylosoxidans NBRC 15126	-	-
		Sdo. denitrificans FST	-	-
		D. oestradiolicum DSM 16959	-	-
		S. estronivorans AXB	*F7P65_RS04060*	37.40
		S. bisphenolivorans YL23	*L858_RS0104680*	38.00
	17580	*C. estronivorus*	*MB02_16645*	55.56
		Sphingomonas sp. KC8	*KC8_11780*	37.16
		Vibrio sp. H5	-	-
		A. xylosoxidans NBRC 15126	*AX27061_5422*	56.24
		Sdo. denitrificans FST	*ACG33_13110*	56.28
		D. oestradiolicum DSM 16959	*CBW56_07150*	40.28
		S. estronivorans AXB	*F7P65_RS04055*	38.18
		S. bisphenolivorans YL23	*L858_RS0104685*	37.37
	18530	*C. estronivorus*	*MB02_16645*	55.56
		Sphingomonas sp. KC8	*KC8_11780*	36.04
		Vibrio sp. H5	-	-

		A. xylosoxidans NBRC 15126	*AX27061_1570*	35.60
		Sdo. denitrificans FST	*ACG33_13110*	56.34
		D. oestradiolicum DSM 16959	*CBW56_07150*	39.86
		S. estronivorans AXB	*F7P65_RS04055*	36.75
		S. bisphenolivorans YL23	*L858_RS0104685*	37.90
Phasin (PhaP)	*12100*	*C. estronivorus*	*MB02_01750*	76.65
		Sphingomonas sp. KC8	*KC8_11785*	45.22
		Vibrio sp. H5	-	-
		A. xylosoxidans NBRC 15126	*AX27061_1570*	45.53
		Sdo. denitrificans FST	*ACG33_13110*	36.95
		D. oestradiolicum DSM 16959	*CBW56_RS07120*	43.73
		S. estronivorans AXB	*F7P65_RS04055*	58.40
		S. bisphenolivorans YL23	*L858_RS0104685*	56.88
PHB depolymerase (PhaZ)	*09590*	*C. estronivorus*	*MB02_08585*	81.40
		Sphingomonas sp. KC8	*KC8_18185*	66.67
		Vibrio sp. H5	-	-
		A. xylosoxidans NBRC 15126	*AX27061_1430*	46.32
		Sdo. denitrificans FST	-	-
		D. oestradiolicum DSM 16959	-	-
		S. estronivorans AXB	*F7P65_RS03690*	51.96
		S. bisphenolivorans YL23	*L858_RS0106030*	48.12
PHB oligomer hidrolase (PhaY)	*15075*	*C. estronivorus*	*MB02_05805*	80.27
		Sphingomonas sp. KC8	*KC8_00605*	57.95
		Vibrio sp. H5	-	-
		A. xylosoxidans NBRC 15126	*AX27061_3624*	32.11
		Sdo. denitrificans FST	-	-
		D. oestradiolicum DSM 16959	*CBW56_09945*	26.95
		S. estronivorans AXB	*F7P65_RS02785*	58.74
		S. bisphenolivorans YL23	*L858_RS0108480*	58.74

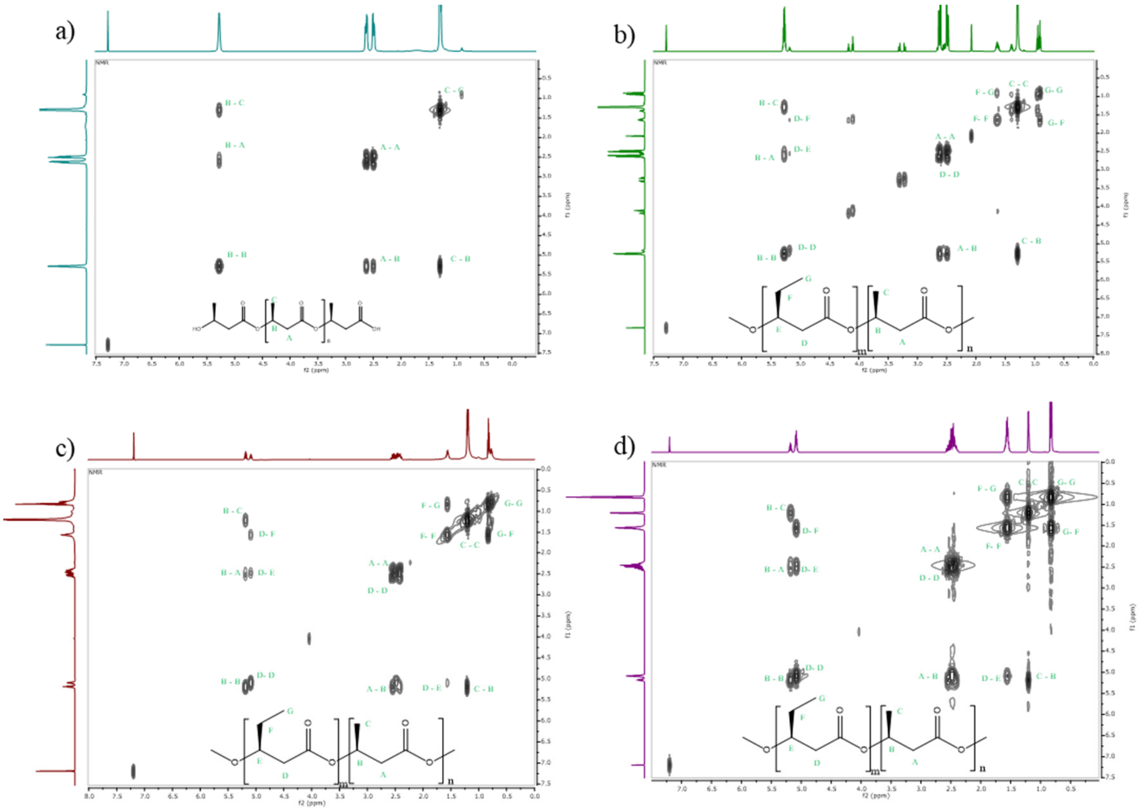

Figure S1. ^{1}H-^{1}H Correlation Spectroscopy (COSY) analysis of the PHA copolymer produced by *C. tardaugens* NBRC 16725. (a) PHB commercial standard, (b) PHBV commercial standard, (c) PHBV formed in M63 0.1 N minimal medium supplemented with 2 mM E2. (d) PHBV formed in M63 0.1 N minimal medium supplemented with 1.89 mM TES. The correlation between protons are shown in the spectrum.
